# Prevalence and factors associated with depression and suicidal ideation among people with diabetes mellitus and hypertension in Uganda

**DOI:** 10.4314/ahs.v24i3.40

**Published:** 2024-09

**Authors:** Rahel Nkola, Mark Mohan Kaggwa, Moses Muwanguzi, Moses Kule, Godfrey Zari Rukundo, Scholastic Ashaba

**Affiliations:** 1 Department of Psychiatry, Faculty of Medicine, Mbarara University of Science and Technology, Uganda; 2 African Centre for Suicide Prevention and Research, Mbarara, Uganda; 3 Bugando medical centre, Mwanza Tanzania; 4 Faculty of Medicine, Mbarara University of Science and Technology, Uganda; 5 Department of Psychiatry, Mbarara Regional Referral Hospital, Mbarara, Uganda

**Keywords:** Depression, suicidal ideation, diabetes mellitus, hypertension, Uganda, sub-Saharan Africa

## Abstract

**Background:**

Depression and suicide ideation are more common among patients with chronic physical illness including diabetes mellitus (DM) and hypertension (HTN). Depression is often undetected, underdiagnosed, and undertreated during routine care and may complicate into suicidal ideation in this population. This study aimed to determine the prevalence of depression and suicidal ideation, and associated factors among people living with DM and/or HTN in Uganda.

**Methods:**

We enrolled 512 participants and assessed depression using PHQ-9, and suicidal ideation using item 9 of PHQ-9. We run logistic regression models to determine the factors associated with depression and suicidal ideation among those with DM only, HTN only or both.

**Results:**

The overall prevalence of depression and suicidal ideation was 22.07% and 10%, respectively. Among participants with both DM&HTN the prevalence of depression was 26.3% while 30.4% had comorbid suicidal ideation. Fear of complications (AOR = 7.21; 95% CI =2.68-19.39; p = 0.01) was significantly associated with depression. Adherence on antidiabetic medications (AOR = 0.10; 95% CI = 0.02-0.72; p = 0.02) was protective against depression.

**Conclusion:**

The prevalence of depression and suicidal ideation among patients with DM and/or HTN in Uganda is high.

## Introduction

In Uganda the prevalence of diabetes mellitus (DM) and hypertension (HTN) is estimated at 26.4% and 1.4% respectively^1^, and is anticipated to increase by 166.9% by 2035^2^. People with DM and/or HTN in Uganda have poor health seeking behavior, lack health education, and many resort to using herbal medicine^3^, which results in many of them presenting to the health facility at a late stage of the disease with a lot of complications including depression and suicidal ideations^3^, ^4^. Depression is the most common mental health problem and second cause of disability worldwide after cardiovascular diseases (CVDs) in the general population^5^. There is an established increase of comorbid depression among people with Non – Communicable Diseases (NCDs) especially DM and HTN in lower and middle income countries (LMIC)^6^. People with DM and HTN have been identified to be at a higher risk of depression and suicidal ideation^7^-^9^. The 12-month prevalence of comorbid depression with NCDs worldwide is between 9.3% and 23.0%^10^. Depression affects one in three people with hypertension^11^, and one in four people with diabetes mellitus^12^. Systematic reviews and meta-analyses have shown evidence of a high prevalence of depression among people with DM and HTN^13^, ^14^. A study in the United States estimated the prevalence of depression among people with comorbid DM and HTN at 13.2%^15^. Depression among people with DM and HTN is reported to be underdiagnosed and under-treated in Uganda^16^. Moreover, depressive symptoms may increase the risk of suicidal ideation among people with DM and HTN^17^.

Suicidal ideation is common among people with DM and HTN^18^-^21^. A study in Australia reported a 14% prevalence of suicidal ideation among people with diabetes^22^. Moreover, in a Nigerian study at the endocrinology and cardiology clinics of the University of Nigeria teaching hospital, involving 530 people 18-64 years diagnosed with hypertension and diabetes, the estimated prevalence of suicidal ideation at 6.3% among people with diabetes and 7.8% among people with essential hypertension^23^. Moreover, suicidal ideation contributes to poor treatment outcomes, increasing mortality and morbidity among affected people^24^, ^25^. However, sparse data on suicidal ideation among people with DM and HTN has been reported in East African countries including Uganda.

Among people with DM and HTN the onset of depression and suicidal ideation may manifest during the time of receiving a diagnosis or while undergoing treatment with the struggle to control blood pressure and maintain normal blood glucose levels^26^. Associations between co-morbid depression in DM and HTN can be bidirectional where by one condition can cause the other and vice versa^27^. On the other hand, multiple factors including fear of complications (such as neuropathy, retinopathy and kidney diseases), history of alcohol use and smoking cigarettes, being widow/widower, and poor social support play an important role in promoting and reinforcing depression and suicidal ideation among individuals with DM and/or HTN^25^, ^28^, ^29^. Furthermore, the presence of DM and/or HTN in a person doubles the odds of comorbid depressive disorder while the presence of depressive symptoms makes the afflicted person more vulnerable to develop HTN or DM^27^, ^30^. However, depression is curable if it is identified and treated early and appropriately^31^. Early treatment of depression limits psychiatric complications especially suicidal ideations and physical complications of DM and HTN such as neuropathy, retinopathy, kidney diseases, and loss of eye site^29^. However, the prevalence of depression and suicidal ideation among people with diabetes mellitus and hypertension in rural Uganda has not been studied. Therefore, this study aimed to determine the prevalence and factors associated with depression and suicidal ideation among people with DM and or HTN in rural south western Uganda.

## Methods

### Study design and setting

This was a cross-sectional quantitative study among people with DM and/or HTN attending the Diabetes and Cardiology Clinic at a tertiary hospital in southwestern Uganda. The study included people aged 18 years and above with a diagnosis of either DM, HTN, or both, enrolled in care for at least 6 months.

### Study participants

Participants were individuals aged 18 years and above with a diagnosis of diabetes and/or hypertension for at least 6 months who were attending the diabetes and hypertension clinic at Mbarara regional referral hospital during the study period.

### Sampling procedure

Convenience sampling was used until the required sample size was reached. Participants were approached by the research assistant following their review by the clinicians in the respective clinics. They were given information about the study including the benefits of participating in the study and the risks that may be associated with their participation. They were assured of their privacy and confidentiality as participants in the study and they were informed that their participation in the study was voluntary. They were given a chance to ask questions for clarity before making any decision to participate in the study. Those who showed their willingness to participate in the study were taken to a private room where further information was provided after which they were asked to sign a written informed consent before they were enrolled in the study. After signing a consent form, each participant then responded to a questionnaire that captured varied information including sociodemographic information including age, sex, level of education, marital status, family history of mental illness, history of cigarettes smoking, history of alcohol use, current blood pressure, and current random blood sugar levels. We also collected information on depression, suicidal ideation, social support, fear of complications, and adherence to medicines.

### Eligibility criteria

*Inclusion criteria:* We included individuals aged 18 years and above, who have been with DM and/or HTN for at least 6 months attending the diabetic and cardiology clinics at MRRH who provided a written informed consent to participate in the study.

*Exclusion criteria:* We excluded potential participants who were physically ill and weak to withstand the length of the interview as well as those having DM and/or HTN for less than 6 months (in order to prevent making diagnosis of acute distress and adjustment reaction due to receiving the diagnosis). People with active symptoms of mental illness, including hallucinations and delusions that could have interfered with their ability to concentrate and comprehend the purpose of the study and the contents of the consent form and questionnaire were also excluded.

## Study Measures

### Patient Health Questionnaire -9 (PHQ-9)

Depression among the study participants was assessed using the nine-item Patient Health Questionnaire (PHQ-9). The PHQ-9 is a self-administered questionnaire with a sensitivity and specificity of 88% for major depressive disorder at a cutoff of 10 (32). The PHQ-9 uses a Likert type scale where for every answer, not at all = 0, several days = 1, more than half the days = 2, and nearly every day = 3. The PHQ-9 has been used in Uganda and Ethiopia with excellent psychometric properties^33^-^35^. The PHQ-9 also categorizes depression in terms of severity whereby 1-4 = minimal depression, 5–9 = mild depression, 10–14 = moderate depression, 15–19 = moderately severe depression, and 20–27 = severe depression^36^. A cut-off score of 10 was used to determine whether participants had depression or not.

### Suicidal ideation

We adopted a question item 9 from the phq-9 to assess for suicidal ideation. Which asks “over the last 2 weeks how often have you been bothered by any of the following problems”? “Thoughts that you would be better off dead or of hurting yourself in some way.” This question has a Likert type response where for every answer not at all = 0, several days = 1, more than half the days = 2, and nearly every day = 3. A score of one and above on this item was used to indicate the presence of suicidal ideation in the past two weeks as used in other studies in Uganda^37^. In this study the tool had good reliability with a Cronbach's alpha of 0.91.

### Oslo social support Scale-3

Social support among the study participants was assessed using the Oslo social support Scale-3. The Oslo social support scale-3 is a three-item scale that provides a brief measure of social support and it is considered to be one of the best predictors of mental health. It covers different fields of social support by measuring the number of people the respondent feels close to, the interest and concern shown by others, and the ease of obtaining practical help from others^38^. This scale has been previously used in low income countries^39^, and its use has been validated in Nigeria with good internal consistency (Cronbach's alpha = 0.50)^38^. The scale has also been used among adult patients with tuberculosis in Kenya with a Cronbach's alpha of 0.91^40^. The Oslo social support scale-3 scores range from 3-14 with a score of 3 - 8 = poor support; 9 – 11 = moderate support; and 12 – 14 = strong support. In the present study the Cronbach's alpha was 0.74.

### Fear of complications questionnaire

Fear of complications was assessed using the fear of complication questionnaire. The fear of complication questionnaire was developed by Taylor et al (2005) and provides a brief measure of fear of complications^41^, ^42^. The fear of complications questionnaire covers different aspects of perceived fear of complications such as fear of kidney diseases, eye sight problems, heart attack, problem of circulation, and stroke among others. The fear of complications questionnaire has been reported to have excellent reliability with a Cronbach's alpha of 0.94^42^. The tool has been used in similar settings in Ethiopia^28^. The questionnaire includes 15 items of which each item is scored by the following points: 0 for “no fear”, 1 for “minor fear”, 2 for “moderate fear”, and 3 points for “greatest fear”. All 15 items are added up making a total score of 0 - 45 points. A higher score implies higher levels of fear. A score of 30 and above is considered as clinically meaningful and relevant fear^41^. In this study a cut-off of 30 was used. The Cronbach's alpha of FCQ in this study was 0.99.

### The Adherence in Chronic Disease Scale (ACDS)

Adherence to medicines was assessed using the adherence in chronic diseases scale (ACDS). The ACDS includes 7 questions with sets of five suggested answers to each question. Depending on the answer each item of the scale is awarded 0 – 4 points. A score of more than 26 points reflects high adherence to treatment, scores of 21 – 26 reflect medium adherence, while less than 21 reflect low adherence. According to the validation study the ACDS questionnaire has a satisfactory level of reliability with a Cronbach alpha of 0.75)^43^. It has been used in many chronic illnesses such as diabetes mellitus, hypertension, chronic heart diseases^43^.

### Random blood glucose levels

Current Random blood glucose levels (RBG) were recorded. RBG of ≤7.5mmol/l were considered normal and that of >7.5 were considered abnormal

### Blood Pressure (BP in mmHg)

Blood pressure was measured using a digital sphygmomanometer with an appropriate cuff size on the patients' right arm in the seated position with feet on the floor after a five-minute rest. Then the systolic and diastolic blood pressures were recorded respectively.

### Ethical Considerations

The study received ethical approval from the research ethics committee of Mbarara University of Science and Technology (MUSTREC #2021-243) and clearance from Uganda National council for Science and Technology (#HS1818ES). All participants provided written informed consent before enrollment in the study and each participant received a stipend of 5000 Uganda shillings to compensate for their time. Participants who had depression and suicidal ideation were referred to the department of psychiatry for further assessment and treatment.

### Data Analysis

STATA version 16.0 was used for data analysis. Means and standard deviations were used to summarize continuous variables while percentages and frequencies were used to summarize categorical variables. Chi square tests were used to determine the differences between the participants who had depression and those that had suicidal ideation. Two separate logistic regression models were run to determine the factors associated with depression and suicidal ideation. A p<.05 for the significance level was considered at a 95% confidence interval [CI].

## Results

### Participant characteristics

A total of 512 participants were included in the analysis and 37.89% of the participants had both DM and HTN, 25.20% had DM alone, while 36.9% had HTN alone. The mean age of the participants was 57.7 (SD±12.69) years. Majority of the participants were women 378 (77.83%) and 23 (4.49%) of the participants had fear of complications. The overall prevalence of depression at PHQ-9 cut off score of 10 was 22.07%. Among participants with both DM and HTN the prevalence of depression was 26.3%, 12.4% among those with DM alone, and 24.3% among those with HTN alone. The overall prevalence of suicidal ideation among participants was 10%, among those with DM 7.8%, 11.1% among those with HTN and 10.3% among those with both DM and HTN (Table 1). The severity of depression (overall) was: minimal (46.29%, n = 237/512), mild (31.64%, n = 162/512), moderate (16.6%, n = 85/512), moderately severe (4.69%, n = 24/512), and severe depression (0.78%, n = 4/512) (Table 1).

**Table 1:** Characteristics of the sample, stratified by chronic disease comorbidity

	Total (N = 512)	DM (n = 129)	HTN (n = 189)	HTN &DM (n = 194)
	Mean (SD) or Frequency (%)	Mean (SD) or Frequency (%)	Mean (SD) or Frequency (%)	Mean (SD) or Frequency (%)
*Age*	(57.74 ± 12.69)	(51.25 ± 13.02)	(59.77 ± 13.05)	(60.09 ± 10.51)
*Sex*				
Male	134 (26.2)	34 (26.4)	49 (25.9)	51 (26.3)
Female	378 (73.8)	95 (73.6)	140 (74.1)	143 (73.7)
*Education level*				
None	138 (26.9)	28 (21.7)	59 (31.2)	51 (26.3)
Primary	250 (48.8)	64 (49.6)	92 (48.9)	94 (48.5)
Secondary	80 (15.6)	21 (16.3)	27 (14.3)	32 (16.5)
Tertiary	44 (8.7)	16 (12.4)	11 (5.8)	17 (8.7)
*Marital status*				
Single/divorced	61 (11.9)	16 (12.4)	30 (15.9)	15 (7.7)
Married/cohabiting	315 (61.5)	89 (68.9)	107 (56.6)	119 (61.3)
Widow/widower	136 (26.6)	24 (18.6)	52 (27.5)	60 (31.0)
*Employment status*				
Employed	108 (21.1)	33 (25.6)	31(16.4)	44 (22.7)
Unemployed	404 (78.9)	96 (74.4)	158 (83.6)	150 (77.3)
*Family history of mental illness*				
No	423 (82.6)	108 (83.7)	159 (84.1)	156 (80.4)
Yes	89 (17.4)	21 (16.3)	30 (15.9)	38 (19.6)
*History of cigarette smoking*				
No	461 (90.0)	120 (93.0)	169 (89.4)	172 (88.7)
Yes	51 (10.0)	9 (7.0)	20 (10.6)	22 (11.3)
*History of alcohol use*				
No	400 (78.1)	109 (84.5)	141 (74.6)	150 (77.3)
Yes	112 (21.9)	20 (15.5)	48 (25.4)	44 (22.7)
*Current Blood pressure*				
Normal	50 (9.8)	28 (21.7)	8 (4.2)	14 (7.2)
Abnormal	462 (90.2)	101 (78.3)	181 (95.8)	180 (92.8)
*Current Random blood glucose level*				
Good control	157 (44.8)	48 (37.2)	30 (100.0)	79 (40.7)
Poor control	196 (55.5)	81 (62.8)	-	115 (59.3)
*Antidiabetics medication adherence*				
Low	207 (40.4)	9 (7.0)	189 (100.0)	9 (4.6)
Medium	124 (24.2)	51 (39.5)	-	73 (37.6)
High	181 (35.4)	69 (53.5)	-	112 (57.7)
*Anti-hypertensive medication adherence*				
Low	177 (34.6)	129 (100.0)	17 (9.0)	31 (16.0)
Medium	93 (18.2)	-	45 (23.8)	48 (24.7)
High	242 (47.2)	-	127 (67.2)	115 (59.3)
*Social support*				
Poor	189 (37.0)	47 (36.4)	67 (35.5)	75 (38.7)
Moderate	202 (39.4)	47 (36.4)	84 (44.4)	71 (36.6)
Strong	121 (23.6)	35 (27.2)	38 (20.1)	48 (24.7)
*Fear of complications*				
No fear	489 (95.5)	121 (93.8)	189 (100.0)	179 (92.3)
Fear	23 (4.5)	8 (6.2)	-	15 (7.73)
*Depression*				
No	399 (77.9)	113 (87.6)	143 (75.7)	143 (73.7)
Yes	113 (22.1)	16 (12.4)	46 (24.3)	51 (26.3)
*Suicide ideation*				
No	461 (90)	119 (92.2)	168 (88.9)	174 (89.7)
Yes	51 (10)	10 (7.8)	21 (11.1)	20 (10.3)

### Relationship between depression and other variables

Individuals with depression were statistically significantly older than those without depression (61.3[±13.6] vs. 56.7 [±12.3] years, t = -3.5, p = 0.001). Depression was higher among the individuals who (i) smoked cigarettes (43.1% vs. 19.5%, X2 =17.5, p<0.001), (ii) used alcohol (34.8% vs. 18.5%, X2 =13.6, p<0.001) compared to those who did not. There was statistically significant differences between participants with depression and those without depression in terms of marital status (X2 =11.8, p0.003), adherence on DM medications (X2 =12.8, p =0.002), adherence on HTN medications (X2 =23.4, p<0.001), chronic disease diagnosis (X2 = 9.6, p = 0.008), and fear of complication (X2 =21.1, p = <0.001) (Table 2).

**Table 2:** Socio-demographic and clinical characteristics of the participants stratified by depression and suicidal ideation

Study variables	Depression			Suicidal ideation		
	No (n = 399)	Yes (n = 113)	t^2^/*X*^2^ (*p*-value)	No (n = 461)	Yes (n = 51)	t^2^/X^2^ (*p*-value)
						
*Age*	56.7 ± 12.3	61.3 ± 13.6	-3.5 **(0.001)**	57.7 ± 12.8	58.4 ± 12.1	-0.4 (0.71)
*Sex*						
Male	107 (79.8%)	27 (20.2%)	0.4 (0.53)	123 (91.8%)	11 (8.2%)	0.6 (0.43)
Female	292 (77.2%)	86 (22.8%)		338 (89.4%)	40 (10.6%)	
*Education level*						
None	100 (72.4%)	38 (72.4%)	5.2 (0.16)	123 (89.1%)	15 (10.9%)	1.6 (0.66)
Primary	195 (78.0%)	55 (22.0%)		223 (89.2%)	27 (10.8%)	
Secondary	66 (82.5%)	14 (17.5%)		75 (93.8%)	5 (6.2%)	
Tertiary	38 (86.4%)	6 (13.6)		40 (90.9%)	4 (9.1%)	
*Marital status*						
Single/divorced	55 (90.2%)	6 (9.8%)	11.8 **(0.003)**	55 (90.2%)	6 (9.8%)	3.5 (0.18)
Married/cohabiting	250 (79.4%)	65 (20.6%)		289 (91.8%)	26 (8.2%)	
Widow/widower	94 (69.1%)	42 (30.9%)		117 (86.0%)	19 (14.0%)	
*Employment status*						
Employed	85 (78.7%)	23 (21.3%)	0.04 (0.83)	99 (91.7%)	9 (8.3%)	0.4 (0.53)
Unemployed	314 (77.7%)	90 (22.3%)		362 (89.6%)	42 (10.4%)	
*Family history of mental illness*						
No	335 (79.2%)	88 (20.8%)	2.3 (0.13)	385 (91.0%)	38 (9.0%)	2.6 (0.11)
Yes	64 (71.9%)	25 (28.1%)		76 (85.4%)	13 (14.6%)	
*History of cigarette smoking*						
No	372 (80.5%)	90 (19.5%)	17.5 **(<0.001)**	414 (89.8%)	47 (10.2%)	0.3 (0.59)
Yes	28 (54.9%)	23 (45.1%)		47 (92.2%)	4 (7.8%)	
*History of alcohol use*						
No	326 (81.5%)	74 (18.5%)	13.6 **(<0.001)**	358 (89.5%)	42 (10.5%)	0.6 (0.44)
Yes	73 (65.2%)	39 (34.8%)		103 (92.0%)	9 (8.0%)	
*Current Blood pressure*						
Normal	40 (81.6%)	9 (18.4%)	0.4 (0.51)	43 (87.8%)	6 (12.2%)	0.3 (0.57)
Abnormal	359 (77.5%)	104 (22.5%)		418 (90.3%)	45 (9.7%)	
*Current random blood glucose level*						
Good control	247 (76.7%)	75 (23.3%)	0.8 (0.39)	286 (88.8%)	36 (11.2%)	1.4 (0.23)
Poor control	152 (80.0%)	38 (20.0%)		175 (92.1%)	15 (7.9%)	
*Antidiabetics medication adherence*						
Low	153 (73.9%)	54 (26.1%)	12.8 **(0.002)**	184 (88.9%)	23 (11.1%)	0.5 (0.76)
Medium	89 (71.8%)	35 (28.2%)		113 (91.1%)	11 (8.9%)	
High	157 (86.7%)	24 (13.3%)		164 (90.6%)	17 (9.4%)	
*Anti-hypertensive medication adherence*						
Low	146 (82.5%)	31 (17.5%)	23.35 **(<0.001)**	163 (92.1%)	14 (7.9%)	1.4 (0.51)
Medium	55 (59.1%)	38 (40.9%)		82 (88.2%)	11 (11.8%)	
High	198 (81.8%)	44 (18.2%)		216 (89.3%)	26 (10.7%)	
*Social support*						
Poor	141 (74.6%)	48 (25.4%)	2.8 (0.25)	167 (88.4%)	22 (11.6%)	1.1 (0.59)
Moderate	158 (78.2%)	44 (21.8%)		183 (90.6%)	19 (9.4%)	
Strong	100 (82.6%)	21 (17.4%)		111 (91.7%)	10 (8.3%)	
*Fear of complications*						
No fear	390 (79.7%)	99 (20.3%)	21.1 **(<0.001)**	444 (90.8%)	45 (9.2%)	7.0 **(0.008)**
Fear	9 (39.1%)	14 (60.9%)		17 (73.9%)	6 (26.1%)	
*Chronic illness*						
Diabetes mellitus	113 (87.6%)	16 (12.4%)	9.6 **(0.008)**	119 (92.2%)	10 (7.8%)	1.0 (0.60)
Hypertension	143 (75.7%)	46 (24.3%)		168 (88.9%)	21 (11.1%)	
Both	143 (73.7%)	51 (26.3%)		174 (89.7%)	20 (10.3%)	
						

### Relationship between suicidal ideation and other variables

The individuals who had fear of complication had suicide ideations compared to those with no fear of complication (26.1% vs. 9.2%, χ2 = 7, p = 0.008) (Table 2).

**Table 2:** Logistic regression of the factors associated with depression

Variables	Bivariate		Multivariable	
	Unadjusted Odds Ratio 95%CI	*p*-value	Adjusted Odds Ratio 95%CI	*p*-value
Age	1.03 (1.01 – 1.05)	**0.001**	1.02 (1.00 – 1.04)	0.09
*Sex*				
Male	1		1	
Female	1.17 (0.72 – 1.89)	0.53	1.42 (0.75 – 2.71)	0.28
*Education level*				
None	1		1	
Primary	0.74 (0.46 – 1.19)	0.22	0.85 (0.48 – 1.49)	0.57
Secondary	0.56 (0.28 – 1.11)	0.10	0.69 (0.31 – 1.56)	0.37
Tertiary	0.42 (0.16 – 1.06)	0.07	0.49 (0.16 – 1.51)	0.21
*Marital status*				
Single/divorced	1		1	
Married/cohabiting	2.38 (0.98 – 5.78)	0.06	3.01 (1.14 – 7.912)	**0.03**
Widow/widower	4.10 (1.64 – 10.26)	**0.003**	4.20 (1.52 – 11.61)	**0.01**
*Employment status*				
Employed	1		1	
Unemployed	1.06 (0.63 – 1.78)	0.83	0.89 (0.47 – 1.68)	0.72
*Family history of mental illness*				
No	1			
Yes	1.49 (0.88 – 2.50)	0.13	1.51 (0.84 – 2.73)	0.17
*History of cigerrate smoking*				
No	1		1	
Yes	3.39 (1.86 – 6.16)	**<0.001**	2.14 (0.99 – 4.65)	0.05
*History of alcohol use*				
No	1		1	
Yes	2.35 (1.48 – 3.74)	**<0.001**	2.04 (1.10 – 3.79)	**0.02**
*Current blood pressure*				
Normal	1		1	
Abnormal	1.29 (0.60 – 2.74)	0.51	0.87 (0.35 – 2.12)	0.75
*Current Random blood glucose level*				
Good control	1		1	
Poor control	0.82 (0.53 – 1.28)	0.39	1.05 (0.57 – 1.95)	0.87
*Antidiabetic medication adherence*				
Low	1		1	
Medium	1.11 (0.68 – 1.84)	0.67	1.00 (0.50 – 2.00)	0.99
High	0.43 (0.25 – 0.74)	**0.002**	0.42 (0.21 – 0.80)	**0.01**
*Antihypertensive medication adherence*				
Low	1		1	
Medium	3.25 (1.85 – 5.73)	**<0.001**	2.34 (1.21 – 4.53)	**0.01**
High	1.05 (0.63 – 1.74)	0.86	0.95 (0.523 – 1.74)	0.89
*Social support*				
Poor	1		1	
Moderate	0.82 (0.51 – 1.31)	0.40	0.78 (0.46 – 1.32)	0.36
Strong	0.62 (0.35 – 1.09)	0.10	0.46 (0.23 – 0.89)	**0.02**
*Fear of complications*				
No fear	1		1	
Fear	6.13 (2.58 – 14.56)	**<0.001**	7.21 (2.68 – 19.39)	**<0.001**

### Factors associated with depression

At bivariate analysis, age, being a widow/widower, history of cigarette smoking, history of alcohol use, high level of adherence on antidiabetic medication, medium level of adherence on antihypertensive medication and fear of complications were statistically significantly associated with depression. However, at multivariable analysis fear of complications (AOR = 7.21; 95% CI =2.68-19.39; p = 0.01), medium level of adherence on antihypertensive medications (AOR = 2.34; 95% CI = 1.21- 4.53; p = 0.01), being married/cohabiting (AOR = 3.01; 95% CI = 1.14-7.912; p = 0.03), being widow/widower (AOR = 4.20; 95% CI = 1.52-11.61; p = 0.01), history of cigarette smoking, (AOR = 2.14; 95% CI = 0.99-4.65; p = 0.05) history of alcohol use (AOR = 2.04; 95% CI = 1.10-3.79; p = 0.02) remained significantly associated with depression. However high level of adherence on antidiabetic medications (AOR = 0.10; 95% CI = 0.02-0.72; p = 0.02) reduced the odds of having depression (Table 3).

**Table 3:** Logistic regression analysis of the factors associated with suicidal ideation

Variables	Bivariate		Multivariable	
	Unadjusted odds ratios (95% CI)	*p*-value	Adjusted odds ratios (95% CI)	*p*-value
Age	1.00 (0.98 – 1.03)	0.71	0.99 (0.97 – 1.02)	0.73
Sex				
Male	1		1	
Female	1.32 (0.66 – 2.66)	0.43	0.93 (0.41 – 2.15)	0.87
*Education level*				
None	1		1	
Primary	0.99 (0.51 – 1.94)	0.98	1.15 (0.56 – 2.38)	0.70
Secondary	0.55 (0.19 – 1.57)	0.26	0.68 (0.20 – 2.10)	0.50
Tertiary	0.82 (0.26 – 2.6)	0.74	1.17 (0.31 – 4.29)	0.82
*Marital status*				
Single/divorced	1		1	
Married/cohabiting	1.49 (0.56 – 3.94)	0.42	0.83 (0.30 – 2.33)	0.74
Widow/widower	0.82 (0.32 – 2.00)	0.69	1.54 (0.52 – 4.54)	0.43
*Employment status*				
Employed	1		1	
Unemployed	1.28 (0.60 – 2.71)	0.53	1.09 (0.47 – 2.51)	0.84
*Family history of mental illness*				
No	1		1	
Yes	1.73 (0.88 – 3.41)	0.11	1.52 (0.75 – 3.08)	0.25
*History of cigerrate smoking*				
No	1		1	
Yes	0.75 (0.26 – 2.17)	0.60	0.82 (0.24 – 2.82)	0.76
*History of alcohol use*				
No	1		1	
Yes	0.74 (0.35 – 1.58)	0.44	0.77 (0.31 – 1.86)	0.57
*Current blood pressure*				
Normal	1		1	
Abnormal	0.77 (0.31 – 1.91)	0.58	0.63 (0.23 – 1.70)	0.37
*Current random blood glucose level*				
Good control	1		1	
Poor control	0.68 (0.36 – 1.28)	0.23	0.61 (0.28 – 1.33)	0.22
*Antidiabetics medication adherence*				
Low	1		1	
Medium	0.78 (0.37 – 1.66)	0.52	1.00 (0.39 – 2.52)	0.99
High	0.83 (0.43 – 1.61)	0.58	0.92 (0.43 – 2.00)	0.84
*Antihypertensive medication adherence*				
Low	1		1	
Medium	1.56 (0.68 – 3.59)	0.29	1.37 (0.545 – 3.41)	0.50
High	1.40 (0.71–2,77)	0.33	1.489 (0.689 – 3.20)	0.3 1
*Social support*				
Poor	1		1	
Moderate	0.79 (0.41 – 1.51)	0.47	0.77 (0.39 – 1.51)	0.73
Strong	0.68 (0.31 – 1.49)	0.34	0.66 (0.28 – 1.53)	0.34
*Fear of complications*				
No fear	1		1	
Fear	3.48 (1.31–9.30)	**0.013**	5.03 (1.67 – 15.13)	**0.004**

### Factors associated with suicidal ideation

The factors which were significantly associated with suicidal ideation at univariate analysis was fear of complications (OR = 3.48, 95% CI,1.31-9.30, p = 0.013). At multivariate analysis fear of complications (OR = 5.03, 95% CI,1.67-15.13, p = 0.004) remained statistically significantly associated with suicidal ideation (Table 4).

**Table 4:** Logistic regression analysis of the factors associated with suicidal ideation

	Bivariate		Multivariable	
	Unadjusted odds ratios (95% CI)	*p*-value	Adjusted odds ratios (95% CI)	*p*-value
Age	1.00 (0.98 – 1.03)	0.71	0.99 (0.97 – 1.02)	0.73
Sex				
Male	1		1	
Female	1.32 (0.66 – 2.66)	0.43	0.93 (0.41 – 2.15)	0.87
*Education level*				
None	1		1	
Primary	0.99 (0.51 – 1.94)	0.98	1.15 (0.56 – 2.38)	0.70
Secondary	0.55 (0.19 – 1.57)	0.26	0.68 (0.21 – 2.10)	0.50
Tertiary	0.82 (0.26 – 2.6)	0.74	1.17 (0.31 – 4.29)	0.82
*Marital status*				
Single/divorced	1		1	
Married/cohabiting	1.49 (0.56 – 3.94)	0.42	0.83 (0.30 – 2.33)	0.74
Widow/widower	0.82 (0.32 – 2.00)	0.69	1.54 (0.52 – 4.54)	0.43
*Employment status*				
Employed	1		1	
Unemployed	1.28 (0.60 – 2.71)	0.53	1.09 (0.47 – 2.51)	0.84
*Family history of mental illness*				
No	1		1	
Yes	1.73 (0.88 – 3.41)	0.11	1.52 (0.75 – 3.08)	0.25
*History of cigerrate smoking*				
No	1		1	
Yes	0.75 (0.26 – 2.17)	0.60	0.82 (0.24 – 2.82)	0.76
*History of alcohol use*				
No	1		1	
Yes	0.74 (0.35 – 1.58)	0.44	0.77 (0.31 – 1.86)	0.57
*Current blood pressure*				
Normal	1		1	
Abnormal	0.77 (0.31 – 1.91)	0.58	0.63 (0.23 – 1.70)	0.37
*Current random blood glucose level*				
Good control	1		1	
Poor control	0.68 (0.36 – 1.28)	0.23	0.61 (0.28 – 1.33)	0.22
*Antidiabetics medication adherence*				
Low	1		1	
Medium	0.78 (0.37 – 1.66)	0.52	1.00 (0.39 – 2.52)	0.99
High	0.83 (0.43 – 1.61)	0.58	0.92 (0.43 – 2.00)	0.84
*Antihypertensive medication adherence*				
Low	1		1	
Medium	1.56 (0.68 – 3.59)	0.29	1.37 (0.545 – 3.41)	0.50
High	1.40 (0.71–2,77)	0.33	1.489 (0.689 – 3.20)	0.31
*Social support*				
Poor	1		1	
Moderate	0.79 (0.41 – 1.51)	0.47	0.77 (0.39 – 1.51)	0.73
Strong	0.68 (0.31 – 1.49)	0.34	0.66 (0.28 – 1.53)	0.34
*Fear of complications*				
No fear	1		1	
Fear	3.48 (1.31–9.30)	**0.013**	5.03 (1.67 – 15.13)	**0.004**

## Discussion

In this cross-sectional study we determined the prevalence of depression and suicidal ideation as well as the associated factors among people with DM and/or HTN attending the diabetes and cardiology clinic at a tertiary hospital in southwestern Uganda. The overall prevalence of depression was 22.07% although the prevalence was significantly higher among participants with both HTN and DM. The factors significantly associated with depression among the study participants were fear of complications, high level of adherence to antidiabetic medications, being married/cohabiting, being widow/widower, history of alcohol use while factors associated with suicidal ideation among study participants was fear of complication.

### Prevalence of depression among study participants

The prevalence of depression among the study participants was higher than what has been estimated in the general population (4.6%) in Uganda^44^. Additionally, this prevalence was higher than what has been estimated in Nigeria at 16.5% using the PHQ-9^45^. The higher prevalence of depression among patients with DM and HTN is likely due to the psychological burden of living with a chronic medical condition in addition to the structural changes in the brain including cerebral atrophy, lacunar infarcts, and blood flow changes due to the hypo- and hyper perfusion related to DM and HTN^46^.

The prevalence of depression among people with DM was lower compared to 43.6% in a study done in Ethiopia^47^. It should be noted that the study in Ethiopia used the Beck Depression Inventory Scale (BDI-II) at 14- cutoff to estimate depression contrary to the use of PHQ-9 in our study. The prevalence of depression among participants who had both DM and HTN was higher compared to 13.2% in a study done in United States. In the study in the United States the DBI-II was used to screen for depression 15, 48, while we used the PHQ-9 and hence the psychometric properties of the two scale may explain the difference in the prevalence. Additionally, the duration of living with HTN and DM, the types of medications used, and the duration of treatment of the participants in the two studies was also different^15^.

The prevalence of depression among participants who had HTN alone was low compared to 33.3% in a study done in Trivandrum-India based on the same tool (PHQ-9) but majority of the participants in the Trivandrum study were largely from the urban setting and had received treatment for at least one year^49^. Moreover a previous study in the same area had documented a higher prevalence of depression among the residents^50^. People who had both DM and HTN had higher prevalence of depression which echoes findings of existing research citing the burden of coping with two chronic conditions^15^. Having both DM and HTN comes with a double burden of struggling to control blood pressure and maintain normal blood glucose levels^26^, ^27^, ^30^.

### Prevalence of suicidal ideation among study participants

The overall prevalence of suicidal ideation was 10 % although the prevalence was significantly higher among participants who had HTN. This prevalence was higher compared to 6.3% and 7.8% for DM and HTN respectively in the study done in Nigeria^19^. This differences could have been attributed to the raised suicidal behaviors during COVID-19 pandemic (the period during which our data was collected)^51^, ^52^. However, the prevalence was lower than 14% in the study done in Australia^18^. The high prevalence of suicidal ideation in Australia is likely due to the higher burden of suicide in the general population (12.5% Australia vs 4.6% in Uganda)^53^, which is beyond the explanations related to DM and/or HTN.

### Prevalence of suicidal ideation among depressed study participants

The prevalence of suicidal ideation among depressed diabetic participants was 30.4% while it was 6.3% among depressed hypertensive participants. Furthermore, the prevalence of suicidal ideation among depressed participants with both diabetes mellitus and hypertension was much higher (23.5%). These high prevalence of suicidal ideation among depressed participants especially those with DM may be due to the fact that depression is the predictor of f suicidal ideation among diabetic patients^54^, and it has also been documented that co -existence of diabetes mellitus and depression increases the risk for suicide ideation than diabetes mellitus alone^18^.

### Factors associated with depression among study participants

Fear of complications and high level of adherence in antidiabetic medications, were significantly associated with depression among the study participants. The significant association between fear of complications and depression found in our study compares to what has been reported in a study in Ethiopia^28^. Fear blocks full participation in life, causes a person to get weaker and lonelier with each passing mealtime. Higher levels of fear lower a person's self-esteem causing a negative view of self and in the long run other complications such as loss of site, renal failure, and neuropathies may occur resulting in depression and other psychological complications^42^. In this study we found that high level of adherence to antidiabetic medications lowered the risk of depression similar what has been documented in previous studies involving patients with diabetes and hypertension^55^-^57^. People with DM and/or HTN who are highly adherent to their medications maintain good control of their blood glucose levels^58^, hence reducing the psychological burden associated with poor glycemic control^59^.

Moreover, being widow/widower was significantly associated with depression among study participants. Our study found that being a widow/widower was highly associated with depression, these findings are in agreement with other studies which reported increased risk of depression among people with diabetes who had lost a spouse^60^, ^61^. Marriage provides care and social support to those with diabetes and hypertension, and family is a significant source of support for those adjusting psychologically to disease which may not be available to widows and widowers. Being a widow/widower is associated with a lot of stress from the grief due to loss of a loved one, thus contributing to the development of depression. Again, our study surprisingly found that being married/cohabiting were significantly associated with depression among study participants contrary to the previous studies. Participation of spouses in the management of a partner's chronic illness has the potential to improve the partner's disease outcomes, adherence to treatment, and ability to adapt to their sickness. Nonetheless, spouses can occasionally be ineffective thus leading to undermining their partners illness hence poor outcomes^62^.

### Factors associated with suicidal ideation among study participants

In this study fear of complications was the only factor associated with suicidal ideation among our study participants. This differs from what has been reported in previous studies that have identified factors such as sex, lack of education, unemployment, family history of mental illness, and poor social support to be associated with suicidal ideation among people with DM and/or HTN^18^, ^19^, ^25^, ^54^

Our findings should be interpreted bearing in mind the following limitations. First, this was a cross-sectional study making it difficult to determine the causal relationship between DM and/or HTN, depression and suicidal ideation. Therefore, multicentre longitudinal studies are required to ably establish this relationship. Secondly, this study was conducted in one tertiary hospital in southwestern Uganda making it difficult to generalize the findings to people with DM and HTN in other parts of the country. Finally, the study did not assess for other factors such as specific medicines taken by the participants and other comorbidities which are likely to be major confounders.

## Conclusions

The present study found higher prevalence of depression and suicidal ideation among patients with DM and/or HTN compared to prevalence of depression in a general population in Uganda. Fear of complications adherence to antidiabetic medications, being married/cohabiting, being widow/widower, and history of alcohol use were factors associated with depression among study participants while factors associated with suicidal ideation among study participants was fear of complication. People with DM and/or HTN should be screened for depression and suicidal ideation for earlier recognition and treatment. Psychosocial assessments aimed to determine psychological challenges of chronic medical conditions should be part of routine clinical evaluation to improve quality of life among patients with DM and HTN.
